# Metabolic Syndrome among Schizophrenic Patients: A Comparative Cross-Sectional Study in the Middle Belt of Ghana

**DOI:** 10.1155/2018/6542983

**Published:** 2018-06-28

**Authors:** Angela Owusu–Ansah, Anto Berko Panyin, Christian Obirikorang, Christian Agyare, Emmanuel Acheampong, Simon Kwofie, Enoch Odame Anto, Emmanuella Nsenbah Batu

**Affiliations:** ^1^Department of Pharmacy Practice, Faculty of Pharmacy and Pharmaceutical Sciences, Kwame Nkrumah University of Science and Technology (KNUST), Kumasi, Ghana; ^2^Department of Molecular Medicine, School of Medical Sciences, Kwame Nkrumah University of Science and Technology (KNUST), Kumasi, Ghana; ^3^Department of Pharmaceutics, Faculty of Pharmacy and Pharmaceutical Sciences, Kwame Nkrumah University of Science and Technology (KNUST), Kumasi, Ghana; ^4^School of Medical and Health Sciences, Edith Cowan University, WA, Australia

## Abstract

The study determined the prevalence of MetS in patients with schizophrenia at the Psychiatric Unit of the Komfo Anokye Teaching Hospital (KATH), Kumasi, Ghana. This comparative cross-sectional study recruited 348 schizophrenic patients comprising 236 antipsychotic-treated and 112 newly diagnosed treatment-*naïve* patients. The MetS prevalence was assessed based on World Health Organization (WHO), International Diabetes Federation (IDF), and the National Cholesterol Education Programme, Adult Treatment Panel III (NCEP ATP III) criteria. The overall prevalence of MetS was 14.1%, 20.4%, and 23.6% using NCEP ATP III, WHO, and IDF criteria, respectively, compared to 7.8%, 3.9%, and 2.2% reported in the general Ghanaian population. The prevalence was significantly higher among treated psychiatric patients compared to treatment-*naïve* group based on NCEP ATP III (17.8% versus 6.2%; p = 0.0001), WHO (26.2% versus 8.0%; p < 0.0001), and IDF (30.3% versus 10.0%; p < 0.0001). MetS was prevalent among patients on atypical antipsychotics compared to typical antipsychotics irrespective of the criteria used (i.e., 17.1% versus 11.1% for NCEP ATP III; 29.5% versus 25.9% for WHO; and 44.3% versus 18.5% for IDF). Using logistic regression model, obesity, raised fasting blood sugar, raised total cholesterol, and decreased high density lipoprotein were observed to be significant predictors of MetS (p<0.05).The study found high prevalence of MetS in Ghanaians with schizophrenia and higher prevalence rate of MetS associated with monotherapy. Regular monitoring of cardiometabolic parameters should be an important therapeutic objective in the management of these patients.

## 1. Introduction

Schizophrenia is a chronic mental disorder which involves disturbances of thought, perception, affect, and social behavior [1]. It is ranked among the top ten causes of disability worldwide with mortality rates two times as high as in the general population [2–4]. Although accidents and suicide account for just a portion of this high mortality rate, more than two-thirds of the rate is caused by different forms of comorbid physical diseases such as cardiovascular and metabolic syndrome [3]. The emergence of these physical diseases has been linked to poor lifestyle (smoking, sedentary lifestyle, and poor diet) and cardiovascular risk factors [5].

Metabolic syndrome (MetS) is a group of disorders which includes central obesity, dyslipidemia, hypertension, abnormal glucose homeostasis, proinflammatory state, and prothrombotic state [6, 7]. The prevalence of MetS seems to be common and rising in the general population possibly due to high intake of calories and a lifestyle of inactivity, irrespective of definition criteria [8, 9]. It is, however, worth considering that some population groups and, for that matter, particular patient groups even have an increased propensity of developing MetS. Several studies have convincingly indicated that schizophrenic patients are twice at risk of developing MetS compared to the general population [8, 10, 11]. Before the introduction of antipsychotics, there were increasing reports of glucose dysregulations (i.e., glucose intolerance, insulin resistance, and hyperglycaemia) by researchers in schizophrenic patients [12]. Moreover, several recent studies have reported increased prevalence of hyperglycaemia, insulin resistance, and impaired fasting glucose tolerance in first episode, antipsychotic-naïve schizophrenic patients compared to healthy control subjects [11].

Consequently, treatment with antipsychotics in people with schizophrenia tends to worsen or even exacerbate these MetS and, thus, contribute to the increased prevalence from 40% to 60% versus 30% in the general population [13, 14]. The atypical antipsychotics are generally known to possess greater propensity of causing these metabolic abnormalities than the typical antipsychotics. Concerning the metabolic dysregulation that accompanied treatment with antipsychotic medications, several studies have recommended a baseline and continuous monitoring (every 6 months) of fasting blood glucose, glycated haemoglobin, and fasting lipid profile to be done in routine clinical practice with all atypical antipsychotics [15–17].

However, in resource constraint health systems as we find in psychiatric hospitals in Ghana, such monitoring is not routinely undertaken, hence the difficulty in determining the prevalence of these metabolic dysregulations. There is a need for current data to serve as a basis for decision-making in terms of routine monitoring for metabolic dysregulations among these patients by healthcare providers in Ghana. Therefore, this study sought to determine the prevalence of MetS among patients with schizophrenia.

## 2. Methods

### 2.1. Study Design and Setting

This was a comparative cross-sectional study which took place at the Out-Patient Department of the Psychiatry Unit of Komfo Anokye Teaching Hospital, (KATH) over a period of five (5) months (April to August 2016). KATH is a 1200-bed facility located in Kumasi in the Ashanti region of Ghana with a projected population of 4,780,380 according to the 2010 census, accounting for 19.4% of Ghana's total population. The Ashanti region is centrally located in the middle belt of Ghana. It lies between longitudes 0.15W and 2.25W, and latitudes 5.50N and 7.46N. The region is divided into 27 districts. The Psychiatry Unit, which is a department under Internal Medicine Directorate, serves both in- and out-patients. About 150 schizophrenic patients are attended to every month on out-patient basis at the Psychiatry Unit.

### 2.2. Study Population and Subject Selection

This study comprised 112 newly diagnosed antipsychotic-naïve and 236 patients on antipsychotics who were receiving treatment at the psychiatric unit. These participants were randomly selected into the study. Laboratory investigations which include fasting blood glucose and lipid levels [triglycerides (TG), low density lipoprotein (LDL), very low density lipoprotein (VLDL), and total cholesterol (TC) as well as measurements of blood pressure and anthropometric measurements (height, weight, waist, and hip circumference)] were carried out.

### 2.3. Inclusion Criteria

 Patients with confirmed diagnosis of schizophrenia according to the International Classification of Diseases (ICD-10) criteria and attending the clinic for review as scheduled by their medical doctors, who were on either monotherapy or dual therapy (fluphenazine-based combination therapies) of antipsychotics, and aged 18 years and above were recruited randomly for the study.

### 2.4. Exclusion Criteria

In-patients and patients with known history of diabetes or hypertension and who were on mood stabilizers and antidepressants/benzodiazepines at the time of enrollment in the study were excluded from the study.

### 2.5. Biochemical Analysis

About 5 ml of venous blood sample was collected after an overnight fast (12–16 hours) and dispensed into fluoride oxalate tubes and vacutainer plain tubes for separation into plasma and serum, respectively. In the laboratory, the samples were centrifuged at 4000 revolution/minute for 5 min within 30 min of collection and stored at -80°C. Assay parameters included fasting plasma glucose (FPG), triglycerides (TG), total cholesterol (TC), low density lipoprotein (LDL), and high density lipoprotein (HDL) cholesterol using the COBAS INTEGRA^(R)^ 400* plus *automated chemistry analyzer.

### 2.6. Anthropometric Measurement

Anthropometric measurements including height (measured without shoes) and weight (to nearest 0.1 kg in light clothing) were taken. Calculation of the Body Mass Indices (BMI) was done by dividing weight (kg) by height squared (m^2^). With respect to the BMI, there were four groupings of subjects: underweight (BMI < 19 Kg/m^2^), normal (BMI between 19 and 24.9 Kg/m^2^), overweight (BMI between 25 and 29.9 Kg/m^2^), and obese (BMI > or = 30 Kg/m^2^). The waist circumference (in centimeter) was measured midway between the lowest rib and the above iliac crest using the Gulick II spring-loaded measuring tape (Gay Mill, WI). The hip circumference was also measured (in centimeters) as the widest circumference around the buttocks. The waist and hip circumference as well as other parameters were used for the calculation of waist to hip ratio (WHR), body adiposity (BAI) [18], and visceral adiposity indices (VAI) [19].

### 2.7. Blood Pressure Measurement

Blood pressure (BP) was recorded after 5 minutes of rest with the subject being in the seated position using manual and an automated sphygmomanometer placed on the subject's right arm. This was measured three times, and the average reading was recorded. Individuals were deemed hypertensive if one or more of these features occur: they were taking antihypertensive medications, they self-reported a diagnosis of hypertension, systolic pressure reading was above 140 mm Hg, or diastolic pressure reading was above 90 mm Hg.

### 2.8. Definition of Metabolic Syndromes

The three most commonly used definitions of metabolic syndrome, as used in this study, are described below.

### 2.9. World Health Organization (WHO) Criteria

The WHO criteria involve the presence of diabetes mellitus, insulin resistance, or impaired glucose tolerance and any two of the following: (1) body mass index (BMI) ≥ 30 kg /m^2^ and/or waist: hip ratio > 0.85 (female), > 0.90 (male); (2) blood pressure, systolic ≥ 140 or diastolic ≥ 90 mmHg or on medication; (3) triglyceride ≥ 1.7 mmol/L and/or HDL-C < 1.01 mmol/L (female), < 0.91 mmol/L (male) [20].

### 2.10. National Cholesterol Education Program, Adult Treatment Panel III (NCEP ATPP III) Criteria

According to the NCEP ATP III, individuals with metabolic syndrome should have at least three of the following: (1) abdominal obesity (waist circumference > 88 cm for women or > 102 cm for men); (2) raised triglyceride (≥ 1.7 mmol/L); (3) low HDL cholesterol (< 1.0 mmol/L in women or < 0.9 mmol/L in men); (4) high blood pressure (systolic BP ≥ 130 mmHg or diastolic BP ≥ 85 mmHg or treatment of hypertension); and (5) raised fasting glucose (≥ 6.1 mmol/L) [21].

### 2.11. International Diabetes Federation (IDF) Criteria

According to the IDF criteria, metabolic syndrome is diagnosed if there is central obesity (waist > 80 cm for women or circumference > 90 cm for men) in addition to any two (2) of the following four (4) factors: (1) triglyceride level ≥ 1.7 mmol/L; (2) HDL cholesterol < 1.29 mmol/L for women or < 1.03 mmol/L for men; (3) blood pressure ≥ 130 /85 mmHg or treatment of previously diagnosed hypertension; and (4) fasting blood glucose (FBG) ≥ 5.6 mmol/L or previously diagnosed type 2 diabetes [20].

### 2.12. Ethical Consideration

The investigations were approved by the Committee on Human Research Publications and Ethics (CHRPE) at School of Medical Sciences, KNUST, Ghana, and the Psychiatry Unit of KATH. Written informed consent was obtained for all participants to maintain their right to self- determination from their visitors/relatives.

### 2.13. Data Analysis

The data obtained were analyzed using Statistical Package for Social Sciences SPSS (version 23.0), statistical packages for Windows. Descriptive statistics that were computed included frequencies and percentages for all categorical variables in addition to means, standard deviations, and ranges for all normally distributed continuous variables, while median and interquartile range were used for continuous variables that were not normally distributed. Differences between groups were carried out using independent sample* t*-test for continuous variable while Chi-square test was used for categorical variables. Univariate binary logistic regression model was used to detect factors associated with MetS among schizophrenia patients. Statistical significance was determined as p value <0.05.

## 3. Results

Subjects in the treatment group were slightly older (with median age of 36 years) than the treatment-naïve group (median age = 32.5 years) but the difference was not significant (p=0.2222). Majority of the study participants were unemployed (55.5%), were single (58.9%), completed Junior High School (45.3%), and had no family history of psychiatric illness (69.1%). Also, several of the study subjects had no history of alcohol intake and smoking (79.1%). Within the antipsychotic-treated group, about half of these subjects (51.3%) had been on a particular medication continuously for more than 12 months** [Table 1]**.

As shown in Table 2, prevalence of MetS was significantly prevalent in subjects on antipsychotic treatment compared to the treatment-naïve patients for all the classifications (p<0.05). Based on the NCEP/ATP III criteria, 17.8% of the patients on treatment had MetS (score ≥3) compared to 6.2% of the treatment-naïve patients with an odd ratio of 11.3 (p=0.0001). Using the IDF criteria, 30.3% of patients on treatment compared to 9.8% of antipsychotic-naïve patients had developed MetS (OR = 12.6, p<0.0001). Similarly, the WHO criteria revealed that 26.2% of the treated patients in comparison with 8.0% of newly diagnosed patients had MetS with an odd ratio of 15.5 (p<0.0001).

The prevalence of MetS was higher in subjects on atypical antipsychotics as compared to those on typical medications for all the classifications, though no significant difference was observed (p>0.05). Furthermore, the risk of developing MetS increased with increasing score of MetS for the all classifications (p>0.05).

It was observed that the odds of MetS increase steadily with increasing bioscore of MetS for the individual classifications. Also, the prevalence of MetS was generally higher in patients on the monotherapy than in those on the dual therapy in all the classifications. There was, however, no statistical difference observed (p>0.05). With respect to the NCEP/ATP III criteria, there were increasing reduced odds of MetS observed with patients on dual therapy with reference to those on monotherapy (OR=0.6). However, no statistical significant difference was observed (p=0.643). Similar observations were seen with the IDF and WHO criteria** [Table 4].**

Using NCEP/ATP III criteria as dependent variable, obesity-WHR, obesity-WHtR, raised fasting blood sugar, raised TC, raised triglycerides, and decreased HDL were significantly associated with increased risks of MetS based on logistic regression model. When the IDF criteria were used as a dependent outcome, age range 50-59 years, obesity-BMI, obesity-WHR, obesity-WHtR, raised FPG, raised TC, raised TG, and raised LDL-C were significant predictors of MetS. Using the WHO criteria as dependent variables, age range 40-49 years, age ≥60 years, obesity-BMI, obesity-WHR, raised TG, and reduced HDL-C were statistically significantly associated with increased risk of MetS** [**S1 and S2**]**.

## 4. Discussion

The prevalence of MetS seems to be common and rising in the general population possibly due to high intake of calories and a lifestyle of inactivity, irrespective of definition criteria [9, 10]. Similar observation was made when the overall prevalence of MetS in this study was compared to the prevalence rate reported from previous study in the general Ghanaian population. Thus, the overall prevalence of MetS among schizophrenic patients in Ghana as determined by IDF (23.6%), WHO (20.4%), and NCEP ATP III (14.1%) in this study was higher compared to the general Ghanaian population prevalence rate (IDF = 7.8%, NCEP ATP III = 3.9% and WHO = 2.2%) as determined by Owiredu et al. [22]. Moreover, a comparison of the overall prevalence from this study to that obtained among psychiatric patients in Ghana shows a consistent rise in the prevalence of MetS among these patients over the years regardless of definition criteria (IDF = 23.6% versus 15.5%; WHO = 20.4% versus 13.5%; NCEP ATP III = 14.1% versus 11.5%) [22, 23]. Furthermore, the MetS prevalence observed in this study was in agreement with a range of prior reports published regionally and worldwide, although there is a considerable variation in criteria and methodology among the published reports [13, 16, 24–26]. However, studies about prevalence rate of MetS are very few in Africa, with most of the studies originating from the southern and northern part of Africa [16, 27–31].

Moreover, the prevalence of MetS was significantly higher among schizophrenic patients on antipsychotic treatment than the treatment-naïve group, which concurs with findings from Saddichha et al. [11] who reported a high prevalence of MetS among antipsychotic-treated group compared with matched healthy control group. Findings from Grover et al. [32]and Shakeri et al. [33] also reported similar findings that MetS was highly prevalent among patients treated with antipsychotics, hence increasing their risk of developing cardiovascular diseases. The smaller prevalence observed in the newly diagnosed treatment-naïve group from the current study further implies that exposure to antipsychotics contributes largely, but not singularly, to the development of cardiometabolic disorders among schizophrenics as well as mentally ill patients.

Among the antipsychotic medications, numerous studies have indicated higher prevalence of MetS associated with atypical antipsychotic use as compared to the typical medications [5, 34]. This is not consistent with the present study; though the current study showed a higher prevalence of MetS in patients on atypical antipsychotics for all the classification criteria compared to the typical drugs, no statistical significant difference was observed [Table 3]. The higher metabolicogenic ability of atypical antipsychotics may be due to their affinity for a wide range of receptor systems (serotonergic, adrenergic, cholinergic, and histaminergic receptors) other than the traditional dopaminergic activity [5]. Several reports have also shown that some atypical antipsychotics have greater propensity of causing metabolic syndrome compared to other medicines within the same class [35–37]. Furthermore, studies by Shirzadi and Ghaemi and Lieberman reported that clozapine and olanzapine treated subjects were at increased risk of weight gain, dyslipidaemias, and hyperglycaemia when compared with other atypical antipsychotics as well as typical medicines. [15, 37]. Therefore, these reports have provided undeniable evidence of the metabolicogenic ability of olanzapine as well as clozapine and permit the inclusion of a black-box warning in prescriptions of these medicines. It is, however, worth noting that strict monitoring of metabolic parameters is paramount for all schizophrenic patients irrespective of antipsychotic used.

Currently, antipsychotic polypharmacy is commonly seen in clinical practice for the management of schizophrenia in most countries as well as Ghana [38]. This practice is not explicitly recommended in most clinical practice guidelines exemplified by National Institute for Health and Care Excellence (NICE), Ghana Standard Treatment Guidelines 2010, and World Federation of Societies of Biological Psychiatry [39]. However, most clinicians use combination therapies in treatment resistant cases or when unsatisfactory response to standard dose of monotherapy is observed [38]. In spite of this, studies that compare the efficacy of mono- and combination therapies have been inconclusive [38]. Furthermore, several cross-sectional studies have reported increased prevalence of metabolic effects and cardiovascular mortality associated with antipsychotic cotreatment [40, 41]. Contrary to these findings, the present study revealed a higher prevalence of MetS associated with monotherapy. Since this study did not consider simultaneous initiation of cotreatment at the beginning of the data collection, it is possible that the adverse effects observed may be related to the direct effect of specific antipsychotic rather than the combination therapy.

Nevertheless, it is unclear whether reports from those previous studies are related to cohort effect, as patients who were selected for antipsychotic combination therapy were psychiatrically and physically sicker than those on monotherapy. Also, such studies mostly considered clozapine- based therapies rather than fluphenazine-based combination therapy as used in this present study [42]. Moreover, this finding could also be explained by the fact that there are more rapid metabolizers in treatment resistant patients. Thus they might experience fewer side effects. Also they might smoke more or use more cannabis, both affecting metabolic syndrome rates. Consequently, it is difficult to assume such risks for all other combination therapies. It is therefore important that longer-term studies of sufficient methodological quality and sample size be conducted to provide sufficient evidence to support the efficacy, long-term safety, mortality, and cost of antipsychotic cotreatment.

The association between age and development of MetS has been widely reported. The prevalence of MetS increases with age in the general population and a similar trend had been generally observed in patients with schizophrenia [17, 33]. Conversely, some studies have also revealed a peak of MetS rates in the third, fourth, or fifth decade of life with subsequent decline in MetS rates in later life [26, 43]. The present study revealed significantly increased risk of MetS in both younger (<49 years) and older (>50 years) age groups as defined by the IDF and WHO classifications. Furthermore, a critical assessment of the significant odds of developing MetS per the IDF and WHO classifications showed that the risk of MetS increased exponentially with increasing age. The increased risk of MetS observed in the younger age group may be attributed to the fact that patients within this group may have a greater predisposition to developing MetS despite being exposed to antipsychotics. Thus, the exposure to antipsychotics may be thought of as quickening MetS onset instead of precipitating it de novo. Also, the significant risk observed in the older ages suggests that, in those groups, age may be a more important risk factor for comorbid MetS as against the neuroleptic prescribed.

From the study, the prevalence of MetS was found to be higher in female subjects compared to the male counterparts but was not statistically significant according to the NCEP ATP III criteria. However, significantly higher prevalence rate was observed in the IDF and WHO classifications. Also, females were approximately 3-4 times at risk of developing MetS compared to the males. These findings agree with a study by You-Kyung et al., [44] who found a significantly higher prevalence rate of MetS in female subjects compared to males according to the IDF classification. However, Sugawara et al. [45] together with other fewer studies [13, 46] have reported slightly higher MetS prevalence rate in men or no significant differences in MetS rates across sexes. The higher prevalence of MetS in females may be attributed to a more sedentary lifestyle, obesity, and age-related physical and hormonal changes [47].

Several studies have suggested that schizophrenia is an independent risk factor for diabetes due to disease-related stress and poor lifestyle of patients [3, 48]. Also, studies have shown that exposure to antipsychotics further predisposes patients to hyperglycaemia, new onset diabetes, and diabetic ketoacidosis in schizophrenic patients with higher risk associated with atypical antipsychotics than the typical medicines [49–51]. Logistic regression model indicates that hyperglycaemia (FPG > 6.4 mmol/L) was significantly associated with higher risks of MetS among patients treated with antipsychotics according to the IDF criteria [Table 6]. These findings concur with previous studies that have identified high prevalence of hyperglycemia and risk of MetS in patients on antipsychotics [49, 52]. Weight gain may be the most obvious signal of MetS and is usually the most distressing issue among patients, thus contributing to noncompliance to antipsychotic medications [53]. Increased abdominal adiposity as measured by waist circumference is important causative risk factor for MetS and a prerequisite for the diagnosis of MetS by the IDF criteria. Results from the logistic regression in this study showed that obesity as defined by BMI, WHR, and WHtR as per the NCEP ATP III, WHO, and IDF criteria were significant predictors of MetS. This shows that both atypical and typical antipsychotics have an almost equal propensity in inducing central obesity (i.e., per definition using WHR) in patients on medication. This is in accordance with reports by Allison et al. and Bobes et al.[14, 54] which revealed that typical antipsychotics and, to a greater extent, atypical medicines are associated with metabolic disturbances and weight gain.

Dyslipidaemia is an important component of the metabolic syndrome and occurs along with glucose dysregulation and weight gain in patients treated with antipsychotics. Logistic regression model revealed that dyslipidaemia (defined as increased TC, TG, and LDL as well as reduced HDL-C) in patients on antipsychotic treatments was associated with increased risk of MetS in all the classifications, though not statistically significant. This agrees with a study by Owiredu et al. [22] which showed that hypertriglyceridaemia and reduced HDL cholesterol were significant risk factors for metabolic syndrome associated with antipsychotic use, especially atypical medicines. In a study to explore the association between hyperglycaemia and antipsychotic use, Haupt and Newcomer [55] suggested that dyslipidaemia as well as increased abdominal obesity impairs glucose metabolism leading to treatment-related hyperglycaemia and type 2 diabetes mellitus. This could therefore explain the significant finding of diabetes observed in patients on antipsychotics treatments in this study.

Notwithstanding, findings in this study are comparable to reports from numerous studies. The study faced some limitations that may affect the generalizability of the results. First, as the study subjects were patients at a single psychiatric hospital, the results obtained from their examination cannot be generalized for the entire population of schizophrenic patients in Ghana. Secondly, variables that can impact MetS, such as physical condition, diet, severity of symptoms, duration of illness, physical activity level, and socioeconomic status, were not adequately evaluated.

## 5. Conclusion

The prevalence of MetS among the psychotic-treated patients in this study is triple that previously reported in nonpsychotic individuals. MetS was also prevalent among patients on atypical and monotherapy than those on typical and dual antipsychotic medications. Regular monitoring of cardiometabolic parameters should be an important therapeutic objective in the management of these patients.

## Figures and Tables

**Table 1:** Sociodemographic characteristics of study participants.

**Sociodemographics**	**Psychotic-treated**	**Treatment-naïve**	**χ** **2,df**	**p-value**
	**n=236**	**n=112**		
**Age (years)**	36.0 (27.0 -45.0)	32.5 (24.8 - 45.3)		0.222
**Age groups (years)**			6.5,3	0.091
< 40	143(60.6)	76(67.9)		
40 – 49	61(25.9)	16(14.3)		
50 – 59	20(8.5)	11(9.2)		
≥ 60	12(5.1)	9(8.0)		
**Gender **				0.647
Male	115(48.7)	58(52.0)		
Female	121(51.3)	54(48.0)		
**Marital status**			10.4,3	**0.015**
Single	139(58.9)	56(50.0)		
Married	79(33.5)	47(4.0)		
Divorced	14(5.9)	2(1.8)		
Widowed	4(1.7)	7(6.2)		
**Employment status**			0.60,2	0.740
Unemployment	131(55.5)	65(58.0)		
Employment	99(41.9)	43(38.3)		
Student	6(2.5)	4(3.6)		
**Educational level**			10.0,4	**0.040**
Primary	12(5.1)	11(9.8)		
JHS	107(45.3)	56(50.0)		
SHS	59(25.0)	18(16.1)		
Tertiary	50(21.2)	18(16.1)		
None	8(3.4)	9(8.0)		
**Family history of Psychotic illness**				0.206
Yes	73(30.9)	27(24.1)		
No	163(69.1)	85(75.9)		
**History of alcohol and smoking**			3.2,3	0.365
Alcohol	20(8.5)	9(8.0)		
Smoking	6(2.5)	0(0.0)		
Alcohol and smoking	22(9.3)	9(8.0)		
None	188(79.7)	94(84.0)		
Duration of Treatment				
< 3 months	23(9.8)			
3 -8 months	44(18.6)			
9 – 12 months	48(20.3)			
>12 months	121(51.3)			

df: degree of freedom; *χ*2: chi-square; n: frequency; JHS: Junior High School; SHS: Senior High School; EPS: extrapyramidal symptoms; p<0.05 is statistically significant.

**Table 2:** MetS among psychotic-treated and treatment-naïve studied subjects.

**Variables**	**Total**	**Psychotic-treated**	**Treatment-naïve**		
	**n=348**	**n=236**	**n=112**	**cOR (95**%** CI)**	**p-value**
**Met / NCEP/ATP III**					
0	75(21.6)	26(11.0)	49(43.8)	1	
1	136(39.1)	93(39.4)	43(38.4)	4.1(2.2-7.4)	**0.0013**
2	88(25.2)	75(31.8)	13(11.6)	10.9(5.1-23.2)	**<0.0001**
≥3	49(14.1)	42(17.8)	7(6.2)	11.3(4.5-47.6)	**0.0001**
**Met / IDF**					
0	47(13.5)	16(6.7)	31(27.7)	1	
1	123(35.3)	71(30.3)	52(46.4)	2.7(1.3 -5.3)	**0.006**
2	96(27.6)	78(32.7)	18(16.1)	8.4(3.8-18.5)	**<0.0001**
≥3	82(23.6)	71(30.3)	11(9.8)	12.5(5.2-30.0)	**<0.0001**
**Met / WHO**					
0	65(18.7)	20(8.5)	45(40.2)	1	
1	122(35.1)	75(31.8)	47(42.0)	3.6(1.9-6.8)	**<0.0001**
2	90(25.9)	79(33.5)	11(9.8)	16.2(7.1-36.8)	**<0.0001**
≥3	71(20.4)	62(26.2)	9(8.0)	15.5(6.5-37.2)	**<0.0001**

NCEP ATP III: National Cholesterol Education Program, Adult Treatment Panel III; IDF: International Diabetes Federation; WHO: World Health Organization; MetS: metabolic syndrome; cOR: crude odds ratio; CI: confidence interval.

**Table 3:** MetS among studied subjects treated with atypical and typical antipsychotics.

**Variables**	**Total**	**Atypical**	**Typical**		
	**n=159**	**n=105**	**n=54**	**OR (95% CI)**	**p-value**
Met / NCEP/ATP III					
0	12(7.5)	6(5.7)	6(11.1)	1	
1	65(41.3)	41(39.1)	24(44.5)	1.7(0.5-5.9)	0.522
2	58(36.2)	40(38.1)	18(33.3)	2.2(0.6-7.8)	0.316
≥3	24(15.0)	18(17.1)	6(11.1)	3.0(0.6-12.9)	0.157
Met / IDF					
0	8(5.0)	4(3.8)	4(7.4)	1	
1	50(31.5)	26(24.8)	24(44.5)	1.1(0.1-9.0)	1.000
2	56(35.2)	44(41.9)	12(22.2)	3.7(0.8-16.9)	0.099
≥3	45(28.3)	31(29.5)	14(25.9)	2.2(0.5-10.2)	0.421
Met / WHO					
0	12(7.6)	6(5.7)	6(11.1)	1	
1	53(33.3)	34(32.0)	19(35.2)	1.8(0.5-6.3)	0.512
2	55(34.6)	36(34.0)	19(35.2)	1.9(0.5-6.7)	0.341
≥3	39(24.5)	29(44.3)	10(18.5)	2.9(0.8-11.1)	0.157

NCEP ATP III: National Cholesterol Education Program, Adult Treatment Panel III; IDF: International Diabetes Federation; WHO: World Health Organization; MetS: metabolic syndrome; OR: odds ratio; CI: confidence interval; p<0.05 is statistically significant.

**Table 4:** MetS among studied subjects on dual and mono antipsychotic therapy.

**Variables**	**Total **	**Dual therapy**	**Monotherapy**	**OR (95**%** CI)**	**p-value**
	**n=236**	**n=77**	**n=159**		
Met / NCEP/ATP III					
0	26(10.9)	14(18.1)	12(7.5)	1	
1	93(39.5)	27(35.1)	66(41.5)	0.4(0.1-0.9)	**0.034**
2	75(31.9)	18(23.4)	57(38.9)	0.3(0.1-0.7)	**0.007**
≥3	42(17.7)	18(23.4)	24(15.1)	0.6(0.2-1.7)	0.643
					
Met / IDF					
0	16(6.7)	8(10.4)	8(5.0)	1	
1	71(30.3)	22(28.6)	49(30.8)	0.5(0.2-1.4)	0.159
2	78(32.7)	22(28.6)	56(35.2)	0.4(0.1-1.12)	0.138
≥3	71(30.3)	25(32.4)	46(28.9)	0.6(0.2-1.6)	0.393
					
Met / WHO					
0	10(8.4)	8(10.4)	12(7.5)	1	
1	38(31.9)	22(28.6)	54(33.9)	0.6(0.2-1.7)	0.418
2	40(33.6)	24(31.2)	55(34.6)	0.7(0.2-1.8)	0.431
≥3	31(26.1)	24(31.2)	38(23.9)	0.9(0.3-2.7)	1.000

NCEP ATP III: National Cholesterol Education Program, Adult Treatment Panel III; IDF: International Diabetes Federation; WHO: World Health Organization; MetS: metabolic syndrome; OR: odds ratio; CI: confidence interval.

## Data Availability

The data used to support the findings of this study are included within the article.

## Supplementary Materials

Supplementary Materials
** S1: Table **5: logistic regression analysis of biochemical parameters predicting risk of MetS among studied subjects.** S2: Table ****6**: logistic regression analysis of age and anthropometric indices predicting risk of MetS among studied subjects.** S3: Table ****7**: prevalence of MetS and components of MetS defined by the NCEP/ATP III, IDF, and WHO criteria stratified by drugs.
