# Supplementary material for: Metabolic Syndrome among Schizophrenic Patients: A Comparative Cross-Sectional Study in the Middle Belt of Ghana
Source: Schizophr Res Treatment. 2018 Jun 28;2018:6542983. doi: 10.1155/2018/6542983 (PMC6046121; doi:10.1155/2018/6542983)
Supplement: Supplementary Materials — S1: Table 5: logistic regression analysis of biochemical parameters predicting risk of MetS among studied subjects. S2: Table 6: logistic regression analysis of age and anthropometric indices predicting risk of MetS among studied subjects. S3: Table 7: prevalence of MetS and components of MetS defined by the NCEP/ATP III, IDF, and WHO criteria stratified by drugs. [file 6542983.f1.docx]

**Table 5: Logistic regression analysis of biochemical parameters predicting risk of MetS among studied subjects**

| Variables | NCEP/ATP III |  | IDF |  | WHO |  |
| --- | --- | --- | --- | --- | --- | --- |
|  | OR (95% CI) | p-value | OR (95% CI) | p-value | OR (95% CI) | p-value |
| FPG/(mmol/L) |  |  |  |  |  |  |
| <6.4 | 1 |  | 1 |  | 1 |  |
| >6.4 | 0.3(0.0-2.3) | 0.25 | 13.0(2.1-81.0) | **0.006** | 3.2(0.6-18.1) | 0.180 |
|  |  |  |  |  |  |  |
| TC/(mmol/L) |  |  |  |  |  |  |
| <6.5 | 1 |  | 1 |  | - | - |
| >6.5 | 4.1(0.3-56.4) | 0.288 | 5.4(0.4-66.3) 0.056 | | - | - |
|  |  |  |  |  |  |  |
| TG/(mmol/L) |  |  |  |  |  |  |
| <1.7 | 1 |  | 1 |  | 1 |  |
| >1.7 | 2.3(0.3-15.2) | 0.406 | 2.5(0.6-10.1) | 0.190 | 5.7(0.1-33.2) | 0.052 |
|  |  |  |  |  |  |  |
| HDL-C/(mmol/L) |  |  |  |  |  |  |
| <1.55 | 1 |  | 1 |  | 1 |  |
| >1.55 | 2.3(0.3-52.7) | 0.152 | 3.1(0.8-12.8) | 0.119 | 2.1(0.4-10.5) | 0.348 |
|  |  |  |  |  |  |  |
| Duration of treatment/(Months) | |  |  |  |  |  |
| <3 | - | - | - | - | - | - |
| 3-8 | 1 |  | 1 |  | 1 |  |
| 9-12 | 2.0(0.1-41.0) | 0.653 | 0.3(0.0-4.0) | 0.372 | 0.9(0.1-12.97) | 0.923 |
| >12 | 1.1(0.1-12.7) | 0.913 | 0.5(0.0-2.5) | 0.411 | 0.9(0.1-5.9) | 0.931 |

Adult Treatment Panel III, IDF = International Diabetes Federation, NCEP ATP III = National Cholesterol Education Program, WHO = World Health Organization, MetS = metabolic syndrome, OR= odds ratio, CI = confidence interval, p<0.05 is statistically significant

**Table 6: logistic regression analysis of age and anthropometric indices predicting risk of MetS among studied subjects**

|  |  |  | |  |  |  |
| --- | --- | --- | --- | --- | --- | --- |
| Variables | NCEP/ATP III | | IDF |  | WHO |  |
|  | OR (95% CI) | p-value | OR (95% CI) | p value | OR (95% CI) | p value |
| Age Groups/(years) | |  |  |  |  |  |
| <40 | 1 |  | 1 |  | 1 |  |
| 40-49 | 2.4(0.8-7.1) | 0.102 | 1.7(0.7-4.1) | 0.280 | 2.8(1.1-7.2) | **0.039** |
| 50-59 | 3.0(0.7-13.7) | 0.157 | 4.5(1.1-17.8) | **0.032** | 3.5(0.8-13.6) | 0.094 |
| ≥60 | 1.4(0.1-13.4) | 0.770 | 0.6(0.7-5.5) | 0.651 | 10.0(1.6-60.9) | **0.013** |
|  |  |  |  |  |  |  |
| BMI/(Kgm-2) | |  |  |  |  |  |
| Normal | 1 |  | 1 |  | 1 |  |
| Overweight | 1.4(0.5-4.2) | 0.554 | 1.5(0.6-3.8) | 0.368 | 0.8(0.3-2.2) | 0.681 |
| Obese | 2.9(0.8-10.2) | 0.096 | 3.6(1.2-10.9) | **0.023** | 6.2(2.0-19.6) | **0.002** |
|  |  |  |  |  |  |  |
| WHR |  |  |  |  |  |  |
| Normal | 1 |  | 1 |  | 1 |  |
| Overweight | 0.4(0.1-4.0) | 0.464 | 0.4(0.1-2.1) | 0.218 | 0.4(0.1-1.7) | 0.206 |
| Obese | 4.5(1.5-13.5) | **0.008** | 4.5(1.8-11.0) | **0.001** | 4.5(1.3-15.4) | **0.016** |
|  |  |  |  |  |  |  |
| WHtR |  |  |  |  |  |  |
| Normal | 1 |  | 1 |  | 1 |  |
| Obese | 5.1(1.4-18.4) | **0.013** | 5.1(1.9-13.6) | **0.001** | 5.0(1.7-14.1) | **0.003** |
|  |  |  |  |  |  |  |
| BAI (%) |  |  |  |  |  |  |
| Underweight | 1 |  | 1 |  | 1 |  |
| Normal | 0.5(0.1-6.2) | 0.618 | 1.3(0.1-14.1) | 0.827 | 1.1(0.1-12.3) | 0.923 |
| Overweight | 0.8(0.0-8.1) | 0.813 | 1.7(0.2-17.3) | 0.674 | 1.1(0.1-11.5) | 0.942 |
| Obese | 0.6(0.1-6.6) | 0.661 | 1.0(0.1-10.5) | 0.976 | 1.0(0.1-10.5) | 0.976 |

Adult Treatment Panel III, IDF = International Diabetes Federation, NCEP ATP III = National Cholesterol Education Program, WHO = World Health Organization, MetS = metabolic syndrome, OR= odds ratio, CI = confidence interval, BMI= Body Mass Index, WHR= Waist-to-hip ratio, WHtR=Waist-to-height ratio, BAI=Body Adiposity Index, p<0.05 is statistically significant

**Table 7: Prevalence of MetS and components of MetS defined by the NCEP/ATP III, IDF and WHO criteria stratified by drugs**

| Variables |  | MetS/ NCEP/ATP III | |  | MetS/ IDF | |  | MetS/ WHO | |
| --- | --- | --- | --- | --- | --- | --- | --- | --- | --- |
|  | Total  (n=236) | Yes (n=49) | No (n=187) |  | Yes  (n=82) | No  (n=154) |  | Yes  (n=71) | No  (n=165) |
| Atypical AP (mono therapy) |  |  |  |  |  |  |  |  |  |
| Olanzapine | 65 | 12(24.5) | 53(28.3) |  | 26(31.7) | 39(25.3) |  | 20(24.3) | 45(27.2) |
| Risperidone | 40 | 9(18.4) | 31(16.6) |  | 11(13.4) | 29(18.8) |  | 13(18.3) | 27(16.4) |
| Typical AP (mono therapy) |  |  |  |  |  |  |  |  |  |
| Haloperidol | 22 | 2(4.1) | 20(10.7) |  | 7(8.5) | 15(9.7) |  | 7(9.9) | 15(9.1) |
| Fluphenazine | 32 | 5(10.2) | 27(14.4) |  | 9(11.0) | 23(14.9) |  | 2(2.8) | 30(18.2) ***** |
| Dual Therapy |  |  |  |  |  |  |  |  |  |
| OF | 30 | 11(22.4) | 19(10.2) |  | 11(13.4) | 19(12.3) |  | 17(23.8) | 13(7.9)***** |
| RF | 22 | 5(10.2) | 17(9.1) |  | 7(8.3) | 15(9.7%) |  | 4(5.6) | 18(10.9) |
| HF | 25 | 5(10.2) | 20(10.7) |  | 11(13.4) | 14(9.1) |  | 8(11.3) | 17(10.3) |

**AP = anti-psychotic, n = frequency, MetS = Metabolic syndrome, OF = Olanzapine + Fluphenazine, RF = Risperidone +Fluphenazine, HF = Haloperidol + Fluphenazine**

**n = number of successfully tested patients**

*** p < 0.05**
